# Effect of α-lipoic acid and exercise training on cardiovascular disease risk in obesity with impaired glucose tolerance

**DOI:** 10.1186/1476-511X-10-217

**Published:** 2011-11-22

**Authors:** Andrea M McNeilly, Gareth W Davison, Marie H Murphy, Nida Nadeem, Tom Trinick, Ellie Duly, Anna Novials, Jane McEneny

**Affiliations:** 1 Sport and Exercise Sciences Research Institute, University of Ulster, Jordanstown, BT37 OQB, UK; 2 Nutrition and Metabolism Group, Queens University Belfast, BT12 6BJ, UK; 3 Southern Eastern Health and Social Care Trust, Ulster Hospital, Dundonald, BT16 IRH, UK; 4 Diabetes and Obesity Laboratory, IDIBAPS (Institut Investigació Biomedica August Pi -Sunyer), and CIBERDEM (Centro de Investigación Biomédica en Red de Diabetes y Enfermedades Metabólicas), Hospital Clinic, Barcelona, Spain

## Abstract

Obese subjects with impaired glucose tolerance (IGT) are more susceptible than healthy individuals to oxidative stress and cardiovascular disease. This randomised controlled investigation was designed to test the hypothesis that α-lipoic acid supplementation and exercise training may elicit favourable clinical changes in obese subjects with IGT. All data were collected from 24 obese (BMI ≥ 30 kg/m^2^) IGT patients. Following participant randomisation into two groups, fasting venous blood samples were obtained at baseline, and before and following intervention. The first group consisted of 12 participants who completed a 12 week control phase followed by 12 weeks of chronic exercise at 65% HR_max _for 30 minutes a day, 5 days per week, while ingesting 1 gram per day of α-lipoic acid for 12 weeks. The second group consisted of 12 participants who completed the same 12 week control phase, but this was followed by 12 weeks of 1 gram per day of α-lipoic acid supplementation only (no exercise). The main findings show a comparatively greater rate of low density lipoprotein (LDL) oxidation in the group consisting of α-lipoic acid only (*p *< 0.05 vs. pre intervention), although total oxidant status was lower post intervention (*p *< 0.05 vs. baseline) in this group. However, exercise and α-lipoic acid in combination attenuates LDL oxidation. Furthermore, in the α-lipoic acid supplement plus exercise training group, total antioxidant capacity was significantly increased (*p *< 0.05 vs. baseline and pre intervention). Body fat percentage and waist and hip circumference decreased following exercise training (*p *< 0.05 vs. post intervention). There were no selective treatment differences for a range of other clinical outcomes including glycaemic regulation (*p *> 0.05). These findings report that α-lipoic acid ingestion may increase the atherogenicity of LDL when ingested in isolation of exercise, suggesting that in IGT the use of this antioxidant treatment does not ameliorate metabolic disturbances, but instead may detrimentally contribute to the pathogenesis of atherosclerosis and development of CVD. However, when α-lipoic acid is combined with exercise, this atherogenic effect is abolished.

## Introduction

It is now recognized that the metabolic disturbances associated with Type 2 Diabetes (T2D) begin prior to the clinical onset of disease [1]. Specifically, subjects with impaired glucose tolerance (IGT) have raised low density lipoprotein cholesterol (LDL-C), triglyceride (TG), insulin, and glucose concentrations and impaired vascular function [2]. Thus, IGT can be viewed as a sub-clinical disease accompanied with an increased risk of cardiovascular morbidity and mortality [3]. Lifestyle interventions including the Diabetes Prevention Program have demonstrated that lifestyle modification is a safe and effective approach to preventing T2D and reducing cardiovascular risk in IGT [4]. For example, there is now strong experimental evidence that exercise training can reduce the risk of developing T2D, through greater weight loss and control of metabolic regulation [5]. However, pharmacological treatment is often preferred as lifestyle interventions are difficult to maintain long term [6]. Whilst pharmacological treatment has been successful in improving the metabolic disturbances associated with IGT, the safety, tolerability and adherence to drugs is similar to T2D, with a significant number of subjects experiencing gastrointestinal stress with metformin, acarbose and orlistat, whereas thiazolidinediones (TZDs) are associated with significant weight gain and oedema [6]. Notably, treatment with rosiglitazone and ramipril is associated with an increased relative risk for heart failure [6].

Due to the side effects associated with some pharmacologic interventions, the use of α-lipoic acid has been considered as a method of reducing the increase in reactive oxygen species (ROS) reported in T2D, which constitute a major causative factor in atherosclerosis and dysregulated glucose metabolism [7]. α-lipoic acid (1,2-dithiolane-3-pentanoic acid (C_8_H_14_O_2_S_2_) is a natural antioxidant found in low concentrations in the mammalian diet, and the most abundant plant sources of α-lipoic acid are spinach, followed by broccoli and tomatoes [8]. It has been reported that α-lipoic acid can increase GLUT-4 translocation, leading to improvements in glycaemic control [9], and this powerful antioxidant may also prevent complications associated with diabetes such as neuropathy and glycation of proteins [9,10]. Moreover, α-lipoic acid is also capable of combating ROS, and could potentially prevent oxidation of LDL-C [11], and decrease atherogenicity and risk of CVD [11]. To the best of our knowledge, no study to date has investigated the role of α-lipoic acid supplementation in combination with moderate intensity aerobic exercise as therapeutic modalities in alleviating the metabolic disturbances associated with IGT. We therefore hypothesise that α-lipoic acid supplementation alone or used in combination with exercise would elicit favourable clinical changes in obese subjects with IGT. We used a randomised controlled experimental design incorporating a comprehensive assessment of cardiovascular risk factors to test this hypothesis.

## Methods

### Subject characteristics

Following approval from a local Research Ethics Committee, twenty four (*n *= 24, 12 males, 12 females) obese (BMI ≥ 30 kg/m^2^, see table 1 for participant characteristics) subjects diagnosed with IGT, following an oral glucose tolerance test (OGTT) [12] were recruited from a local Diabetic Clinic to participant in this study. Participants with a family history of sudden cardiac death or a personal history of cardiovascular or liver complications were excluded from participation, as determined by health history questionnaire. Subjects were also excluded if they smoked, were taking any form of antioxidant supplement, had dyslipidaemia or any type of haematological disorder. All procedures were conducted in accordance with The Declaration of Helsinki.

**Table 1:** Age and physiological characteristics of participant groups

Variable	Group 1 (*n *= 12)	Group 2 (*n=*12)	Combined (*n *= 24)
Age (yrs)	56 ± 8	54 ± 8	54 ± 8
Stature (cms)	1.67 ± 0.1	1.68 ± 0.1	1.47 ± 0.5
Body mass (kg)	87 ± 18	93 ± 14	93 ± 14
Body mass index (kg/m^2^)	32 ± 7	33 ± 6	33 ± 6
Body fat (%)	47 ± 7	50 ± 1	50 ± 1
Waist circumference (cm)	105 ± 23	105 ± 9	105 ± 9
Hip circumference (cm)	110 ± 14	111 ± 13	110 ± 13

All values are mean ± SD. Each group had 6 male and 6 female subjects.

### Experimental design

The present study constituted a balanced, randomised controlled trial.

### Preliminary testing

On arrival of participants at the laboratory, their body mass, stature, body fat percentage (using bioelectrical impedance; Bodystat 1500, Bodystat, UK) and waist and hip circumference were measured at baseline and following intervention using standard methods. A standard OGTT (75 g glucose), was administered to all subjects to confirm impaired glucose tolerance according to clinical guidelines [12]. Systemic arterial blood pressure (BP) was measured in the brachial artery using an Omron M5-1 fully automated BP monitor (Surrey, UK). Three readings were taken at 2 minute intervals following 15 minutes supine rest and the mean value calculated.

### Experimental procedure

Participants were randomly assigned to one of two experimental groups. Subjects in group one (*n *= 12) completed a 12 week control phase during which habitual dietary intake and physical activity was monitored and maintained at consistent levels, followed by 12 weeks of chronic exercise at 65% predicted HR_max _in accordance with ACSM guidelines [13], for 30 minutes a day, 5 days per week, while ingesting 1 g of racemic α-lipoic acid (Cultech Biospeciality Products, Wales) per day for 12 weeks (1 g α-lipoic acid 7 d^.^wk^-1^) [*exercise + α-lipoic acid group*]. This exercise intensity was chosen due to its classification as moderate intensity as outlined by the ACSM [13] and based on the results of other exercise intervention studies in IGT [14,15]. This intensity is the equivalent of a brisk walk in this sedentary population [16]. We assume that all subjects were exercising to the same metabolic cost throughout the 12 week intervention, as all participating subjects were sedentary, thus representing a similar level of baseline fitness prior to the start of the intervention. Following 30 minutes of exercise on a motorised treadmill (Sport Engineering Limited, UK) to acclimatise to the required walking speed, all participants were provided with heart rate monitors (Polar Electro, Finland) and shown how to operate these to ensure they were walking at the required intensity for the duration of the 12 weeks. Subjects in group 2 (*n *= 12) completed a 12 week control phase, followed by 12 weeks of 1 g per day of racemic α-lipoic acid supplementation (1 g α-lipoic acid 7 d^.^wk^-1^) [*α-lipoic acid only group*]. Subjects were asked to refrain from exercise and alcohol consumption for 48 hours prior to all experimental testing, and all testing was conducted between 8.00-10.00 am to control for inter-subject diurnal variation.

### Exercise and supplementation compliance

Subjects were asked to complete weekly exercise diaries detailing total exercise duration, time of exercise, whether exercise was performed in isolation or with family members/friends and perceived feelings following exercise on a Likert scale (-5 = very bad, +5 = very good). Subjects were encouraged to complete walking on flat terrain for the entire duration of the intervention. Compliance with supplementation was determined by pill counts at the end of each 4 week period.

### Haematology

#### Sampling

Venous blood was collected following a 12 hour overnight fast from an antecubital forearm vein following 20 minutes supine rest at baseline, pre-intervention and 12 weeks post intervention using the aseptic technique *via *the Vacutainer method (Becton-Dickinson, Oxford, UK). Blood for total cholesterol, high density lipoprotein cholesterol (HDL-C), LDL-C, TG and homocysteine (Hcy) determination were collected in anaerobic glass vacutainers containing EDTA and immediately placed on ice, while blood for glycosylated haemoglobin (% HbA_1c_), very low density lipoprotein (VLDL), LDL and HDL isolations, total antioxidant capacity (TAC), total oxidant status (TOS) and high sensitivity C-reactive protein (*hs*-CRP) were collected in serum separation glass vacutainers and allowed to clot for 10 minutes in the dark. Blood for glucose determination was collected in sodium fluoride EDTA tubes. After centrifugation at 3000 rpm at 4°C for 10 minutes, the serum/plasma samples were stored in aliquots at -80°C. The cholesterol oxidation products were centrifuged at 3500 rpm for 5 minutes at 4°C and subsequently stored at -80°C. All samples from the same participant were analysed within the same batch.

### Analyses

#### Lipid metabolites

Serum total cholesterol, HDL-C, LDL-C and TG concentration (TG) were measured using methods previously published [17]. Estimates of LDL-C concentration were calculated using the Friedewald formula [18]. The inter-assay CVs for total cholesterol, HDL-C and TGs were 0.7%, 3.0% and 2.3% respectively.

#### Blood glucose and HbA_1c_

Glucose concentration was determined by the immobilised enzyme membrane method in conjunction with a Clark electrode (Leyland Clark, Ohio, USA) on a YSI 2300 analyser (Yellow Springs, USA). The inter-assay CV was < 1%. HbA_1c _was assayed using an Adams A1c HA-8160 analyser (ARKRAY, Japan). The inter-assay CV was < 2.0%.

#### Total homocysteine (tHcy)

tHcy was measured by high-performance liquid chromatography (HPLC) using the method devised by Araki & Sako [19] and modified by Ubbink and colleagues [20]. The inter-assay CV was 2.6%.

#### Lipoprotein isolation and oxidation

VLDL, LDL and HDL lipoproteins were isolated *via *rapid density gradient ultracentrifugation using a Beckman TL100 ultracentrifuge with a fixed angle rotor (TL100.3; Beckman, UK). The procedure was adapted from previously published methodologies [21,22]. Before undergoing oxidation the lipoprotein samples were standardised for protein with PBS. VLDL was standardised to 25 μg/ml, LDL to 50 μg/ml and HDL to 100 μg/ml. Oxidation was mediated by the addition of copper II chloride (CuCl_2_; final concentrations of 35 μM for VLDL, 2 μM for LDL and 5 μM for HDL). The kinetics of oxidation was monitored in a thermostatically controlled spectrophometer at 37°C by measuring absorbance change at 234 nm on a 96 well Spectra Max 190 plate reader. Data was analysed using SoftMax Pro Software Package version 4.8 (Molecular Devices, USA). Following oxidation of each lipoprotein, time at half max (t 1/2max) in minutes, an equivalent measure of oxidation susceptibility as lag time was calculated. The intra and inter-assay CVs were <5%.

#### Total Oxidant Status (TOS)

Serum total oxidant levels were measured using a modification of the method of Erel [23]. Briefly, this assay is based on the assumption that oxidants present in a biological sample can oxidise the ferrous ion-o-dianisidine complex to ferric ion, and the oxidation reaction is enhanced by glycerol molecules. The ferric ion forms a coloured complex with the xylenol orange in an acidic medium, and the intensity of the colour formed by this complex can be measured spectrophotometrically at 560 nm. Unknowns were read against a hydrogen peroxide (H_2_O_2_) standard curve and the results are expressed in terms of μmol H_2_O_2 _equivalent per litre (μmol H_2_O_2 _Equiv/l). The intra and inter-assay CVs were <5%.

#### Total Antioxidant Capacity (TAC)

Serum TAC was measured using a modification of the method of Erel [24]. Briefly, this assay is based on the principle that reduced 2,2'-azinobis-3-ethylbenzothiazoline-6-sulfonic acid (ABTS) molecules are oxidised to the 2,2'-azinobis-(3-ethylbenzothiazoline-6-sulfonic acid radical cation (ABTS^.+^) using H_2_O_2 _in acidic medium. The deep green ABTS^.+ ^radical cations are stable for a longer period of time than ABTS molecules, and when the ABTS^.+ ^molecules are diluted with a more concentrated acetate buffer solution at high pH values, the colour is spontaneously bleached by the antioxidants present in the sample. The bleaching rate is proportional to the concentrations of antioxidants, which is positively related to the TAC of a biological sample. This reaction is spectrophotometrically measured at 660 nm, unknowns were read against a Trolox standard curve. Results are expressed in mmol Trolox Equiv/l. The intra and inter-assay CVs for TAC were <5%.

#### High sensitivity C-reactive protein hs

CRP was measured on an Aeroset analyser (Abbott Laboratories, USA) using a CRP high sensitivity assay kit (Randox laboratories Limited, N Ireland). The formation of an antibody-antigen complex during the assay procedure results in an increase in turbidity, the extent of which is measured as the amount of light absorbed at 550 nm. The CRP concentration was determined from a standard curve. The inter-assay CV was 1.9%.

#### Dietary intake assessment Dietary

intake was assessed at six time points (weeks 0, 6, 11, 12, 18 and 24) using four day unweighed diet diaries completed using a food photography atlas to determine portion size [25]. All dietary information was analysed using Netwisp nutritional analysis programme (Netwisp, Version 3.0, Tinuvel, 2006).

### Statistical analysis

Statistical analysis was performed using the SPSS statistics package (Version 15.0, SPSS, Woking, UK). A prospective calculation of power was performed using the equations of Altman [26]. Data were analysed using parametric statistics following mathematical confirmation of a normal distribution using Shapiro-Wilk *W *tests. Baseline, pre and post intervention data were analysed using a two-way [A *x *B] mixed analysis ANOVA which incorporated one between (group: α-lipoic acid + exercise or α-lipoic acid only) and one within subjects factor (time: baseline, pre and post intervention). When a significant interaction effect was detected, within-participant factors were analysed using Bonferroni-corrected paired sample *t *tests. Between-participant differences were analysed using a one-way ANOVA with a posterior Tukey honestly significant difference test. The alpha level was established at *p *< 0.05 and all values are reported as mean ± SD unless otherwise stated.

## Results

### Exercise and supplementation compliance

All subjects completed the 12 weeks of exercise training and supplementation, thus representing a return of 100% for compliance.

### Energy intake and composition

There were no significant differences in energy and micronutrient composition within or between groups (Table 2).

**Table 2:** Mean dietary intake during control (weeks 0-12) and experimental phase (weeks 12-24) for both groups

	*Week 0*	*Week 6*	*Week 11*	*Week 12*	*Week 18*	*Week 24*
***α-lipoic acid + exercise group (n = 12)***						
Total energy (kcal)	1884 (466)	1909 (472)	1892 (616)	1838 (306)	1965 (366)	2002 (324)
Carbohydrate (g)	223 (70)	219 (67)	215 (77)	205(67)	225(65)	227 (58)
Protein (g)	85 (21)	82 (17)	80 (17)	80 (17)	82 (14)	90 (16)
Total fat (g)	71 (24)	74 (25)	74 (33)	71 (14)	78 (14)	74 (17)
Saturated fat (g)	25 (12)	27 (12)	28 (15)	25 (9)	26 (11)	26 (10)
PUFA (g)	13 (3)	13 (5)	12 (4)	13 (6)	15 (6)	14 (5)
MUFA (g)	23 (8)	23 (7)	23 (8)	22 (5)	24 (5)	24 (5)
Cholesterol (mg)	304 (129)	298 (161)	290 (159)	265 (174)	329 (114)	312 (133)
Alcohol (g)	11 (18)	10 (16)	10(16)	7 (13)	5 (13)	8 (17)
***α-lipoic acid only group (n = 12) ***						
Total energy (kcal)	2525 (982)	2684 (1088)	2506 (855)	2525 (982)	2684 (1088)	2506 (855)
Carbohydrate (g)	293 (139)	331 (139)	320 (100)	293 (139)	331 (139)	320 (100)
Protein (g)	104 (51)	112 (65)	95 (32)	104 (51)	112 (65)	95 (32)
Total fat (g)	101 (40)	107 (46)	94 (49)	101 (41)	107 (46)	94 (49)
Saturated fat (g)	34 (16)	38 (19)	35 (22)	34 (16)	38 (19)	35 (22)
PUFA (g)	19 (11)	19 (12)	15 (10)	19 (11)	19 (12)	15 (10)
MUFA (g)	34 (14)	33 (14)	29 (11)	34 (14)	33 (14)	29 (11)
Cholesterol (mg)	299 (221)	255 (175)	249 (177)	299 (221)	255 (175)	249 (177)
Alcohol (g)	2 (6)	11 (41)	9 (22)	2 (6)	11 (41)	9 (22)

Values are means ± SD.

### Body composition and blood pressure

There was a significant decrease in body fat and in waist and hip circumference in the group undergoing α-lipoic acid + exercise when compared to baseline and pre intervention values (*p *< 0.05 vs. post intervention; Table 3). Systolic and diastolic blood pressure did not change as a function of time or group (*p *> 0.05; Table 3).

**Table 3:** Biochemical and haemodynamic measures at baseline, pre and post intervention for both groups

*Group*	*α-lipoic acid + exercise* *(n = 12)*			*α-lipoic acid only* *(n = 12)*		
*Time *	*Baseline *	*Pre intervention *	*Post intervention*	*Baseline*	*Pre intervention *	*Post intervention*
Body mass (kg)	87.7 (18.7)	87.3 (18.9)	85.5 (17.4)	93.1 (14.3)	93.1 (14.3)	92.4 (14.0)
BMI (kg/m^2^)	31.6 (6.7)	31.4 (6.9)	30.7 (6.4)	32.8 (5.8)	32.8 (5.8)	32.5 (5.7)
Total body fat (%)	46.6 (6.9)	41.6 (5.5)	41.5 (5.4)**†**	49.8 (11.6)	49.4 (11.2)	49.3 (11.8)
Waist circumference (cms)	105.8 (23.3)	100.6 (13.9)	96.1 (13.9)**†**	104.9 (9.0)	104.9 (9.0)	102.7 (9.2)
Hip circumference (cms)	109.9 (13.9)	108.8 (16.0)	103.1 (13.1)**†**	112.5 (12.7)	112.6 (12.8)	111.1 (12.9)
Total cholesterol (mmol**^.^**L^-1^)	5.5 (1.0)	5.5 (0.6)	5.2 (1.0)	4.7 (1.2)	4.7 (1.2)	4.8 (1.1)
HDL cholesterol (mmol**^.^**L^-1^)	1.2 (0.2)	1.3 (0.2)	1.1 (0.1)	1.1 (0.3)	1.1 (0.3)	1.1 (0.2)
LDL cholesterol (mmol**^.^**L^-1^)	3.3 (0.9)	3.4 (0.7)	3.2 (0.9)	3.0 (1.1)	3.0 (1.1)	3.1 (1.1)
Triglycerides (mmol**^.^**L^-1^)	1.9 (1.1)	1.7 (0.7)	1.8 (0.8)	1.6 (0.7)	1.6 (0.7)	1.7 (0.7)
*hs-*CRP (mg/l)	6.2 (5.4)	6.0 (5.7)	6.3 (7.5)	4.6 (5.0)	4.6 (5.0)	4.6 (5.1)
Homocysteine (μmol/l)	11.7 (6.8)	11.2 (3.6)	11.7 (5.1)	12.1 (4.5)	12.1 (4.6)	12.2 (4.6)
Glucose (mmol**^.^**L^-1^)	5.9 (0.9)	5.7 (0.6)	5.6 (0.7)	6.1 (1.0)	6.1 (1.0)	6.0 (0.8)
% HbA_1c_	5.9 (0.3)	5.9 (0.3)	5.7 (0.4)	6.0 (0.5)	6.0 (0.5)	6.2 (1.1)
Systolic BP (mmHg)	143.0 (26.2)	139.2 (20.6)	141.5 (16.9)	141.4 (12.7)	143.2 (12.8)	136.2 (15.4)
Diastolic BP (mmHg)	90.5 (11.6)	88.3 (11.2)	90.3 (11.9)	88.9 (8.6)	90.7 (10)	89.6 (12.7)

Values are means ± SD. **† **indicates within group differences as a function of time from baseline (*p *< 0.05)

### Oxidation of lipoproteins

There was a comparatively greater rate of LDL oxidation (ox-LDL) as a function of α-lipoic acid supplementation only (*p *< 0.05 vs. pre intervention; Table 4), while LDL oxidation was unaffected following α-lipoic acid and exercise. Furthermore, the oxidation potential of VLDL and HDL were unaffected by intervention in both groups (*p *> 0.05).

**Table 4:** Oxidised VLDL, LDL and HDL (time 1/2 max, minutes) at baseline, pre and post intervention for both groups

*Group*	*α-lipoic acid + exercise* *(n = 12)*		*α-lipoic acid only* *(n = 12)*			
*Time *	*Baseline *	*Pre intervention *	*Post intervention *	*Baseline *	*Pre intervention*	*Post intervention*
ox-VLDL	193.2 (6.3)	202.5 (14.5)	205.5 (15.0)	231.5 (12.0)	240.6 (17.1)	229.7 (12.1)
ox-LDL	113.5 (5.0)	114.9 (5.2)	111.7 (3.7)	114.2 (2.5)	118.0 (3.5)	112.7 (2.6)†
ox-HDL	56.0 (4.6)	52.1 (3.4)	56.3 (4.0)	44.8 (1.7)	43.6 (1.5)	42.7 (1.2)

Values are means ± SD. **† **indicates within group differences as a function of time from baseline (*P *< 0.05)

### TOS and TAC

Although TOS was unaffected by α-lipoic acid and exercise treatment (p > 0.05) it was found to be lower post intervention (*p *< 0.05 vs. baseline) in the group randomised to α-lipoic acid supplementation (Table 5). Additionally, TAC was significantly increased in both the α-lipoic acid and exercise group and the α-lipoic acid only group (*p *< 0.05 vs. baseline and pre intervention, for both groups; Table 5).

**Table 5:** Total Oxidant Status (TOS) and Total Antioxidant Capacity (TAC) at baseline, pre and post intervention for both groups

*Group*	*α-lipoic acid + exercise* *(n = 12)*			*α-lipoic acid only* *(n = 12)*		
*Time *	*Baseline *	*Pre intervention *	*Post intervention *	*Baseline *	*Pre intervention*	*Post intervention*
TOS (μmol H_2_0_2 _Equiv/L)	37.0 (8.9)	38.5 (8.2)	35.1 (5.6)	49.7 (9.1)	50.0 (9.5)	38.5 (7.2)†
TAC (mmol.l^-1 ^Trolox Equiv/l)	1.4 (0.2)	1.4 (0.1)	1.8 (0.3)†	1.3 (0.3)	1.3 (0.3)	1.6 (0.4)†

Values are means ± SD. **† **indicates within group differences as a function of time from baseline and pre intervention (*p *< 0.05)

### Lipids, hs-CRP and homocysteine

Total cholesterol, HDL-C, LDL-C, TGs (mmol/l), *hs-*CRP (mg/l) and tHcy (μmol/l) all remained unchanged throughout in both groups (*p *> 0.05 for all; Table 3).

### Glucose and glycosylated haemoglobin (% HbA_1c_)

Table 3 shows the effect of exercise and α-lipoic acid supplementation on blood glucose concentration and HbA1c. There were no differences in blood glucose or in % HbA1c following 12 weeks of α-lipoic acid supplementation with or without exercise (*p *> 0.05).

## Discussion

Strong experimental evidence indicates that α-lipoic acid is beneficial for glycaemic regulation [27], in addition to acting as a powerful dual phase antioxidant *in vivo *[8]. The primary purpose of this research was to ascertain the effects of α-lipoic acid ingestion and 12 weeks of exercise training on glycaemic regulation and lipoprotein oxidation in obese subjects with IGT. This study demonstrates that α-lipoic acid ingestion increases the atherogenicity of LDL cholesterol, suggesting that in IGT, the use of this antioxidant treatment does not ameliorate metabolic disturbances, but instead may detrimentally contribute to the pathogenesis of atherosclerosis and development of CVD. However, when α-lipoic acid was combined with exercise, this atherogenic effect was abolished, postulating that moderate intensity exercise training may attenuate LDL oxidation. Moreover, exercise in combination with α-lipoic acid significantly decreases total body fat and waist and hip circumference. However, α-lipoic acid supplementation alone or in combination with moderate intensity exercise was not effective in improving glycaemic regulation, as determined by peripheral blood glucose and glycosylated haemoglobin.

Despite no change in HDL or VLDL oxidation and notwithstanding that total oxidant status decreased with α-lipoic acid supplementation, the latter demonstrating moderate antioxidant capability, this study reports that oxidation of LDL cholesterol was significantly decreased (as shown by a decrease in time 1/2 max) following α-lipoic acid supplementation, suggesting that α-lipoic acid may be functioning as a pro-oxidant, increasing LDL atherogenicity and promoting atherosclerosis. This isolated finding is supported by studies using animal models to demonstrate that α-lipoic acid and or dihydrolipoic acid may increase the atherogenicity of LDL cholesterol [28]. Reported mechanisms for this effect include the generation and formation of free radical molecular species, and the superoxide anion is highlighted to be one of the main protagonists in the initiation of LDL oxidation [29]. Furthermore, the pro-oxidant activity of α-lipoic acid has previously received attention perhaps due its ability to react with oxidants and subsequently produce other free radical species that may propagate a radical chain reaction and oxidative damage to cells [30].

While α-lipoic acid supplementation in isolation seems to increase LDL oxidation, and thus susceptibility to CVD in IGT subjects, α-lipoic acid ingestion in combination with exercise training abolishes this pro-oxidant effect. The benefits of exercise training in the prevention of T2D and CVD are well documented, with a recent meta-analysis documenting favourable changes to blood pressure, systemic lipids, vascular function and glucose control [31]. Likewise, exercise training protects vulnerable cells against oxidation by an improved enzymatic antioxidant defence network, involving superoxide dismutase [32], where the up-regulation of isoforms of this enzyme is activated by the transcription factors nuclear factor kappaB and mitogen-activated protein kinase [32]. There is a possibility that exercise training protects against peripheral LDL oxidation in IGT subjects, by a beneficial adaptation and improved enzymatic antioxidant network, however, as these molecular properties were not quantified in this work, this hypothesis is speculative.

The notion that α-lipoic acid contributes towards a beneficial adaptation in glycaemic control was reported by Estrada *et al*., [33], where they demonstrated that α-lipoic acid stimulates glucose transport *via *GLUT proteins within the insulin signalling pathway. Although α-lipoic acid supplementation adversely affected LDL oxidation in this investigation, this dual phase antioxidant in addition to exercise had no effect on glycaemic regulation. This finding was surprising given that both exercise and α-lipoic acid are independently associated with an increase in glucose uptake and the reversal of insulin insensitivity in T2D [34]. An intriguing explanation for the lack of glucose related metabolic change with α-lipoic acid ingestion may involve the optimal administered dose of α-lipoic acid. In the present study a 1 g dose of racemic α-lipoic acid (*R- *and *S*- enantiomer) was administered daily for the duration of the experiment, and we chose this amount based on the work of Evans *et al.*, [35] where they demonstrated that 0.9 g of racemic α-lipoic acid for 7 d^.^wk^-1 ^for 6 weeks followed by 1.2 g 7 d^.^wk^-1 ^for 6 weeks significantly decreased plasma fructosamine by 10% (313 to 282 mmol/l) in T2D subjects. In addition, 1 g of α-lipoic acid per day is also considered the maximum daily oral dose achievable without experiencing side effects such as nausea and gastrointestinal stress [35]. Some subjects however, did experience feelings of nausea during the first week of supplementation which then diminished. It is possible that 1 gram of racemic α-lipoic acid per day as used for 12 weeks in this study was not sufficient to produce favourable improvements in glycaemic regulation in systemic IGT blood. Further aggressive treatment with intravenously administered α-lipoic acid may be required to prevent the metabolic disturbances associated with insulin resistance. For example, Sergermann *et al.*, [36] successfully demonstrated that IV administration of α-lipoic acid at a rate of 7.5 mg/(100 g body weight^.^d) produced a 45% decrease in plasma triglyceride concentration, but no improvement in glycaemic control and this was attributed to the racemic composition of the α-lipoic acid mixture. Whilst the positive metabolic actions of α-lipoic acid in improving insulin sensitivity have been reported predominantly in animal models [8], the *R*-enantiomer appears to display a greater effect than the *S*-enantiomer [33]. Therefore, perhaps the racemic mixture used in our investigation was insufficient to elicit favourable changes in metabolic regulation.

Similar to the lack of effect on glucose regulation with α-lipoic acid supplementation, there was no antioxidant effect on LDL oxidation observed with α-lipoic acid, but in fact increased LDL oxidation. α-Lipoic acid possess the ability to scavenge a number of ROS whilst also interacting with other important antioxidants such as ascorbic acid, α-tocopherol and glutathione in order to produce favourable cardiovascular benefits [11]. The availability of *in-vivo *antioxidants such as ascorbic acid and α-tocopherol may explain why α-lipoic acid produced no direct beneficial antioxidant effect at preventing LDL oxidation. Prior to intervention in the α-lipoic acid group, total antioxidant capacity was relatively low compared to values observed for normoglycaemic individuals (1.34-1.40 mmol.l^-1 ^Trolox Equiv/l *vs*. 1.50-1.77 mmol.l^-1 ^Trolox Equiv/l in healthy individuals; Nadeem 2010, unpublished observations). However, following intervention, total antioxidant capacity was significantly improved, and this is consistent with other work demonstrating that a high dose of antioxidants can favourably increase systemic total antioxidant capacity [24].

One plausible explanation for a low total antioxidant capacity at baseline, prior to intervention, is poor dietary intake. Obesity is characterised by increased intake of macronutrients (specifically carbohydrate and fat), and lower than average consumption of fruit and vegetables rich in antioxidants [37]. By examining the dietary intake of subjects, it appears that whilst unweighed diet diaries can provide some useful insight into dietary intake and habits, they are open to considerable bias. It is likely that under reporting of macronutrient intake, or over estimating fruit and vegetable consumption may have occurred in this population. For example, reported energy intake for subjects varies from 1884-2506 kcals/day, which would seem unlikely in obese individuals with a mean body mass of 93 kg. Similarly, some subjects reported a moderate fruit and vegetable consumption (3-4 portions per day), which did not correspond to individual total antioxidant capacity measurements. Ideally, dietary intake would be assessed *via *food photography and picture plate waste (PPW) which has been successfully used to avoid the issue of under reporting/over estimating and is analogous to visual observation [38]. Nonetheless, a low total antioxidant capacity concentration at baseline, suggests low fruit and vegetable consumption resulting in a reduced availability of dietary antioxidants such as ascorbic acid and α-tocopherol. This would exert a consequential effect on the antioxidant potential of α-lipoic acid and its ability to recycle endogenous antioxidants and reduce α-lipoic acid to its oxidised metabolite, dihydrolipoic acid [39]. This is supported by results from Kagan *et al.*, [40] who demonstrated that humans supplementing with high doses of α-tocopherol, may cause a greater recycling of α-tocopherol due to interactions with ascorbic acid. This work supports the hypothesis that antioxidant recycling may be a viable mechanism which may result in decreased oxidation of LDL cholesterol [40]. Therefore, if IGT subjects have compromised *in-vivo *concentrations of primary non-enzymatic antioxidants such as ascorbic acid and α-tocopherol, there is a profound possibility that α-lipoic acid remained in a reduced state, as opposed to being oxidised to dihydrolipoic acid, which is the most effective form for exerting a protective effect on the oxidation of lipoproteins [39].

Moderate intensity exercise training in combination with α-lipoic acid ingestion significantly decreases total body fat and waist and hip circumference. However as no changes were observed for these parameters in the α-lipoic acid only group, it is postulated that these changes were brought about directly by the increased energy expenditure resulting from exercise training, and this has been previously reported in T2D [41]. The close relationship between total and central body fat, cardiovascular disease and hypertension [2] lends further importance to our findings, and the anthropometric changes confirm that in obese IGT subjects, a modest amount of exercise (*i.e *30 mins per day) can increase energy expenditure sufficiently, to favourably reduce body composition and thus decrease susceptibility to CVD.

An obvious limitation of this study is the absence of a normoglycaemic control group; however, the main aim of this work was to examine the effects of α-lipoic acid supplementation in isolation, and in combination with chronic exercise training in obese subjects with IGT, and to this end, all parameters were assessed consistently throughout the 12 week control, and subsequent experimental phase (weeks 12-24). Therefore in essence, the IGT subjects acted as their own control.

In concluding, whilst reports demonstrate that α-lipoic acid may possess antioxidant capabilities to improve glycaemic regulation and modulate the oxidation of lipoproteins [42], the main finding of this investigation shows that LDL atherogenicity may actually be increased with α-lipoic acid supplementation, even in the presence of an enhanced systemic total antioxidant capacity. Our data thus suggests an increased susceptibility to CVD in IGT subjects, which may be controlled to some extent by moderate exercise training. Future work should concentrate on determining whether α-lipoic acid supplementation exerts a pro-oxidant effect on LDL oxidation in the non-diseased state and whether this is attenuated by the addition of exercise. Work is also required to fully ascertain the optimal dose, racemic mixture, and their relationship with exercise in ameliorating the metabolic disturbances associated with insulin resistant conditions such as IGT and T2D.

## Competing interests

The authors declare that they have no competing interests.

## Authors' contributions

AM participated in the experimental design, undertook subject recruitment, data collection and conducted lipoprotein, oxidant and antioxidant analyses. GD conceived of the study, participated in its design, performed the statistical analysis and prepared the manuscript. MM participated in the experimental design and coordination of the study. TT and ED performed standard biochemistry analyses. JMcE and NN facilitated lipoprotein, oxidant and antioxidant analyses. AN facilitated critical discussion and advised on clinical matters associated with the study. All authors read and approved the final manuscript.
